# Preserving avian blood and DNA sampled in the wild: A survey of personal experiences

**DOI:** 10.1002/ece3.9232

**Published:** 2022-08-29

**Authors:** Irene Di Lecce, Joanna Sudyka, David F. Westneat, Marta Szulkin

**Affiliations:** ^1^ Centre of New Technologies University of Warsaw Warsaw Poland; ^2^ Institute of Environmental Sciences Jagiellonian University Kraków Poland; ^3^ Groningen Institute for Evolutionary Life Sciences University of Groningen Groningen The Netherlands; ^4^ Department of Biology University of Kentucky Lexington Kentucky USA

**Keywords:** birds, blood sampling, DNA, long‐term storage, molecular ecology

## Abstract

Collecting and storing biological material from wild animals in a way that does not deteriorate DNA quality for subsequent analyses is instrumental for research in ecology and evolution. Our aims were to gather reports on the effectiveness of methods commonly used by researchers for the field collection and long‐term storage of blood samples and DNA extracts from wild birds. Personal experiences were collected with an online survey targeted specifically at researchers sampling wild birds. Many researchers experienced problems with blood sample storage but not with DNA extract storage. Storage issues generated problems with obtaining adequate DNA quality and sufficient DNA quantity for the targeted molecular analyses but were not related to season of blood sampling, access to equipment, transporting samples, temperature, and method of blood storage. Final DNA quality and quantity were also not affected by storage time before DNA extraction or the methods used to extract DNA. We discuss practical aspects of field collection and storage and provide some general recommendations, with a list of pros and cons of different preservation methods of avian blood samples and DNA extracts.

## Graphical abstract

The collection of biological material from wild animals plays a fundamental role in many disciplines within ecology and evolution and its storage under optimal conditions ensures its usefulness long after its collection. We report results from an online survey, in which we directly asked scientists to share their experiences in the collection and long‐term storage of blood samples and DNA extracts from wild birds. We show that different storage conditions can generate considerable variation in the quality of stored samples, with consequences for research outcomes.

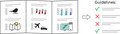

## 1 INTRODUCTION

Ecological and evolutionary processes shaping natural animal populations typically occur over at least multiple years or decades. Consequently, research in ecology and evolutionary biology often requires data extending over long periods of time and records of individuals over their entire life spans (Clutton‐Brock & Sheldon, 2010). Long‐term individual‐based studies have proven extremely valuable in gaining insight into the demographic and life‐history traits of wild populations, for instance by making sense of aspects of breeding, survival, mate choice, and lifetime reproduction (Bouwhuis, 2018; Mills et al., 2015). Over the past three decades, molecular tools have become easier to use and have been widely applied to multiple disciplines such as population ecology/biology (Deyoung & Honeycutt, 2005), biogeography (Riddle et al., 2008), conservation genetics (Primmer, 2009), or behavioral ecology (Bengston et al., 2018). They have also led to the emergence of new fields such as landscape genetics, molecular quantitative genetics, and population genomics (Black IV et al., 2001; Jensen et al., 2014; Manel et al., 2002). These techniques add to the size and richness of biological archives in recent years, spanning several decades and thousands of individuals. The wide array of cost‐efficient molecular tools available nowadays and the increasing computational power able to handle large amounts of data allow researchers to reliably perform a variety of analyses on biological material from wild populations. However, sample storage conditions remain a key issue that can limit the ability to generate high‐quality genetic data from animal blood or other tissues. At the time of logistical planning of a long‐term study, an informed decision concerning sample preservation has to be made. Inadequate preservation might compromise sample quality and research scope, for example, leading to the degradation of nucleic acids (Conrad et al., 2000; Kilpatrick, 2002; Seutin et al., 1991; Zimmermann et al., 2008). In this light, *storage method* and *temperature* are fundamental aspects of sample preservation. Because of their interactive influence on final sample quality, their effects—and limitations—have to be taken into consideration before undertaking sampling for a specific project and ideally taken into account in light of future applications that may arise with the progression of the study.

### 1.1 Storage method

Direct sample freezing is viewed as the method of choice for long‐term storage, since enzymatic and other chemical activities decrease with lower temperatures (Wong et al., 2012). However, other storage methods which require a liquid preservative or physical support (e.g., paper‐based substrate) might provide advantages over freezing, such as a reduction in space and energy consumption, no power outage risks, lower long‐term costs, and easier transfer and shipping of samples. For example, blood can be stored at room temperature in either 95%–100% ethanol, in lysis buffers such as Queen's buffer and Longmire's buffer, or dried on filter paper, such as FTA® cards (Longmire et al., 1997; Seutin et al., 1991; Smith & Burgoyne, 2004). However, such storage methods can differentially impact molecular assays that will be performed on the biological samples. For instance, in a PCR diagnostics study for avian and human malaria, lower accuracy of the test was associated with samples stored in a lysis buffer (containing sodium dodecyl sulfate or SDS) compared with a buffer lacking SDS (Freed & Cann, 2006). SDS may have been the cause since it releases endonucleases and creates extracellular debris. There is also evidence that relative telomere length (RTL) measurements differ significantly depending on storage method: Reichert et al. (2017) showed how RTL of samples stored on FTA® cards at room temperature was significantly shorter than in samples preserved as frozen whole blood or frozen DNA.

### 1.2 Storage temperature

When directly freezing samples, temperatures of −80°C, or as low as possible, are recommended to maximize DNA preservation (Jackson et al., 2012; Wong et al., 2012). In case of biological material collected from birds, storage at −20°C was deemed sufficient to prevent DNA degradation, since avian red blood cell nuclei were considered metabolically inactive (Seutin et al., 1991). However, recent evidence reports that avian blood cells do have functional mitochondria involved in respiratory cellular metabolism (Stier et al., 2013). Consequently, lower storage temperature (e.g., −80°C) may still offer better preservation conditions than freezing at −20°C. When deep‐cold storage is not feasible or practical, samples are stored at room temperature (Kilpatrick, 2002; Seutin et al., 1991; Smith & Burgoyne, 2004). However, multiple studies have reported poor stability of DNA extracted from whole blood samples stored at room temperature and better DNA yields from samples kept at +4°C or lower (Madisen et al., 1987; Richardson et al., 2006; Visvikis et al., 1998). Moreover, filter paper is known to generate different DNA yields depending on storage temperature: Hollegaard et al. (2011) showed how storing dried blood spots (DBS) samples, also known as Guthrie cards, at +4°C negatively affected DNA concentration, which increased when samples were stored at −20°C. Mei et al. (2011) reported similar results on DBS tested for Toxo‐specific immunoglobulin‐M: reduced recovery was observed in DBS stored at room temperature compared with specimens stored at −20°C.

### 1.3 Additional factors influencing sample stability

Technical assessments of specific protocols on DNA degradation (Kilpatrick, 2002; Michaud & Foran, 2011; Seutin et al., 1991; Zimmermann et al., 2008) have considerable value but may fail to capture the impact of some constraints of field collection and long‐term storage on research outcomes. For instance, collecting samples in spring or summer means that biological material may be challenged by high temperatures (even more so in hot climates), with negative consequences on DNA quality/integrity. Access to equipment in the field, such as a fridge or a freezer, may have a positive effect on sample preservation, ensuring sample stability before long‐term storage in the laboratory. Different ways of transferring samples from the field to the laboratory might also affect sample integrity if samples are exposed to high temperatures during transportation or experience delays in shipping. Moreover, storing samples as blood or DNA extracts might have different outcomes on DNA integrity in the long run, so during logistical planning, it might be necessary to take into consideration storage time before DNA extraction. For instance, Schröder and Steimer (2018) found significantly lower DNA extraction yields as well as higher methylation levels of blood samples stored in EDTA at different temperatures after 10 months of storage, relative to samples processed immediately after sampling.

In this light, we asked scientists directly about their perceived assessment of the efficacy of their storage procedures and about what experiences have influenced their decisions regarding the long‐term storage of blood and DNA. Importantly, personal and first‐hand experiences may shed light on how often problems that arise from sample collection or storage can affect DNA quality, thus impacting the final sample size and the quality of published research.

Here, we present the results from an online survey designed for researchers involved in the collection and storage of blood and DNA from wild birds. We circulated the survey among ecologists and field biologists to (i) review practices commonly used for field collection and storage of avian blood and DNA extracts, (ii) assess if any procedural or methodological gaps exist in current knowledge of avian blood and DNA storage, and (iii) improve existing guidelines for the long‐term storage of avian blood and DNA.

## 2 METHODS

### 2.1 Survey

An online survey entitled “Preserving avian DNA from the wild: Your experience of blood sampling, DNA extraction and storage” was created on the Survey Monkey platform (Appendix S1). It comprised three sections (blood sample collection, blood storage, and DNA extraction and storage) with 52 questions, mostly multiple choice. Fourteen of these were open questions, where more detailed answers were required. Out of the 52 questions, 25% (*n* = 13) were designed with a Likert scale, here a five‐point rating scale which allows respondents to express how much they agree or disagree with a particular statement (Derrick & White, 2017). The survey was disseminated from summer 2018 until early spring 2019, specifically targeting researchers working with wild birds in the fields of evolution, ecology, and conservation biology. The survey was advertised on social media using Twitter, by email to colleagues known to have collected avian samples with kind requests for further forwarding, on evolution and ecology international and national mailing lists and dissemination Websites (EvolDir, EvolFrance, the Ornithological Societies of North American newsletter, and zmihor.blogspot.com) and during conferences (i.e., International Society for Behavioural Ecology 2018, International Ornithological Congress 2018 and Polish Evolutionary Conference 2018). Furthermore, to increase response rate from all over the world, the survey was specifically emailed to more than 500 researchers working with wild birds outside Europe and North America, found through searches on the Web of Science. Participation in the survey was anonymous, but respondents could leave their contact information.

### 2.2 Data analysis

Data analysis was carried out in R 4.1.0 (R Core Team, 2022). Chi‐squared tests of independence were used to test the relationship between having experienced storage issues and problems with obtaining adequate DNA quality (yes/no) or quantity (yes/no). Storage issues (i.e., problems with storage which might have negative consequences on DNA integrity) were coded as a yes/no variable. Fisher's exact and Chi‐squared tests of independence were used to test the relationship between storage issues and having changed storage methods for blood samples (yes/no) or DNA extracts (yes/no). Because it is known that filter paper leads to DNA degradation (Hollegaard et al., 2011), a Fisher's exact test was run to specifically investigate this assumption by creating an additional variable from the open answers regarding DNA degradation for the different storage methods. Fisher's and Chi‐squared tests were also used to investigate the relationship between problems with obtaining adequate DNA quality (yes/no) or quantity (yes/no) and several aspects of sample collection and preservation. The investigated aspects were as follows: season of blood sampling (tested as two separate explanatory variables with a separate test: either coded in four categories—Spring, Summer, Autumn, and Winter—or coded as two categories—Dry and Wet season—as respondents could choose only one option), access to equipment in the field (yes/no), means of transportation of blood samples to the laboratory (six categories: Airplane, Car, Courier, Boat, Train, and Other), storage temperature of blood samples (five categories: Room temperature, +4°C, −20°C, −80°C, Other), type of molecular analysis performed on the samples (12 categories: Gene expression, Methylation assay, MHC characterization, Microsatellite assay, Molecular sexing, mtDNA analysis, Parasite DNA analysis, RAD sequencing, SNP chip, SNP genotyping, Telomere length, and Whole‐genome sequencing) and DNA extraction method (six categories: Ammonium acetate, Chelex, Commercial column kit, In‐house protocol, Phenol‐chloroform, and Other). To test whether some storage methods of blood samples were more likely to be associated with DNA quality/quantity problems further downstream, generalized linear models assuming quasibinomial error distribution (to correct for overdispersion) were employed. Occurrence of problems with obtaining adequate DNA quality (yes/no) or quantity (yes/no) was fitted as response variables and storage method for blood samples (seven categories: Ethanol, Lysis buffer, Direct freezing, Filter paper, TE buffer, RNA*later*, and Other) as fixed categorical explanatory variable. Similar models were run to test whether storage time before DNA extraction mattered, with occurrence of problems with obtaining adequate DNA quality (yes/no) or quantity (yes/no) fitted as response variables and storage time (four categories: Up to 6 months, Up to 1 year, More than 1 year, No standard time frame) as fixed categorical explanatory variable. Here, generalized linear models were employed, instead of mixed models (accounting for respondent id as random effect) due to lack of convergence of the mixed models (only 7% of responses were not independent, as the same respondents filled the survey two times or more).

## 3 RESULTS

### 3.1 Blood sample collection

**FIGURE 1:**
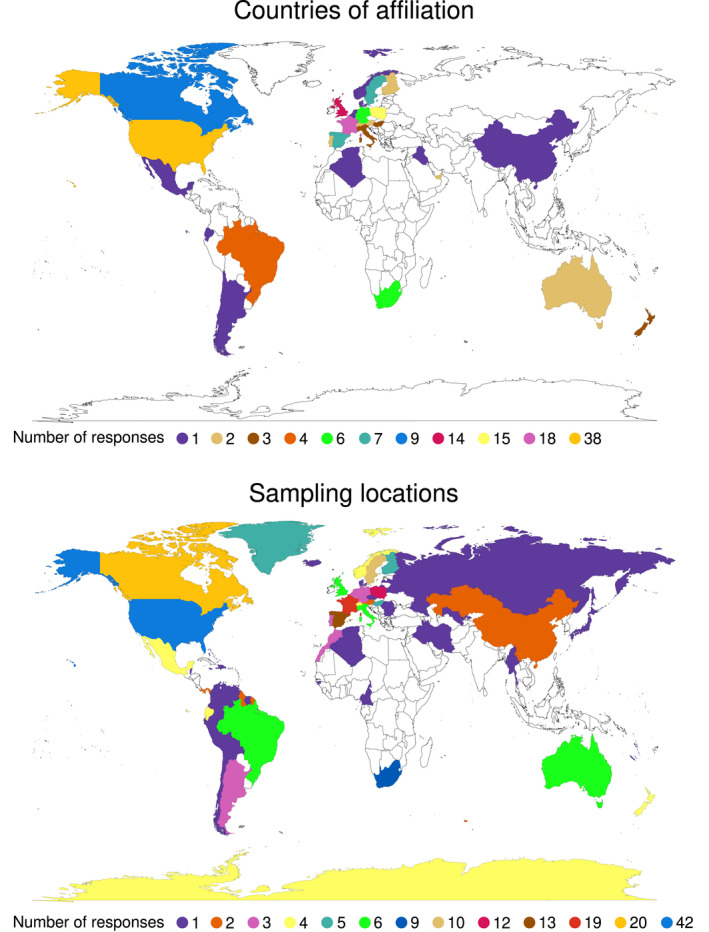
World maps reporting countries of affiliation of respondents and countries where fieldwork and blood sampling occurred.

**FIGURE 2:**
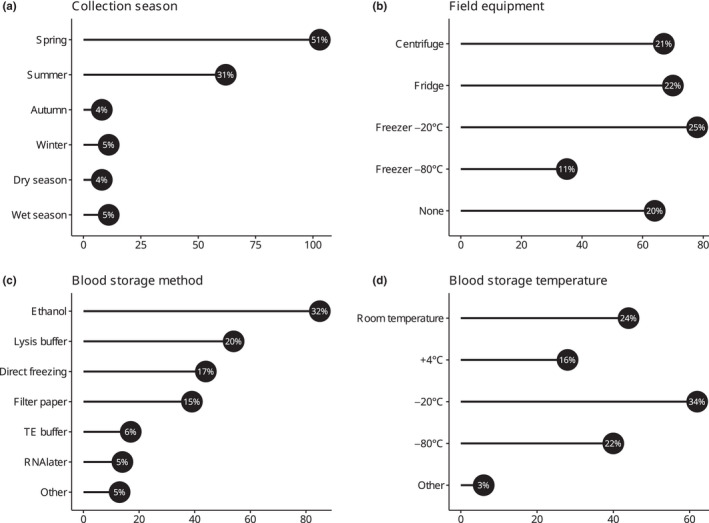
Overview of aspects of collection and preservation of blood samples: Season of blood sampling (a), equipment available in the field (b), storage method (c) and storage temperature for blood samples (d). On the *x* axis, number of responses, with percentages of the total, are shown; note that in (b) and (c) more than one response could be given. “Other” in (c) comprises: EDTA (3 responses), PBS (1), EDTA + PBS (1), a glycerol‐based buffer (1), NBS buffer (1), TNE buffer (2), commercial buffer (3) and heparin buffered tubes (1). “Other” in (d) comprises: −35°C (1 response), −40°C (1), −50°C (1), −70°C (1) and liquid nitrogen (1).

A total of 219 responses to the survey were collected. All anonymized answers are available in Appendix S2. Two hundred and nine responses concerning wild birds and molecular analyses were kept (10 were on domestic species and/or on other types of analyses). Overall, researchers taking part in the survey worked on 123 species of wild birds, encompassing 53 families and 20 orders, with blue tit (*Cyanistes caeruleus*) and great tit (*Parus major*) being the most represented (Tables S1–S3). Of the 155 responses on affiliation, 85% (*n* = 131) were research institutions located in North America and Europe, with the greatest contribution from the United States, France, UK, and Poland, in decreasing order (Figure 1—Countries of affiliation). The experience of respondents in field blood sampling covered 53 countries and territories across the globe, with 63% (*n* = 176) of responses from North America and Europe and the greatest contribution from the United States, France, Canada, Spain, Poland and Sweden, in decreasing order (Figure 1—Sampling locations). Spring and summer were reported in 80% (165/203) of responses to the question “When are blood samples on this project most often collected?” (Figure 2a). Access to equipment in the field, such as a centrifuge, a fridge or a freezer, was reported in 68% (132/195) of responses (Figure 2b shows number of responses, with percentage of the total, for the different types of equipment). Of the 284 multiple‐choice responses to the question “How do you move blood samples from the field site to the permanent laboratory on this project?,” 53% (*n* = 151) were transported by car, followed by airplane (23%; *n* = 64), courier service (11%; *n* = 32), train (6%; *n* = 17), boat (3%; *n* = 9), on foot (2%; *n* = 5), bicycle (1%; *n* = 3), bus (1%; *n* = 2), and one response by helicopter. Of the 204 responses to the question “How large is your sample database on this project?,” 45% (*n* = 91) were between 100 and 1000 blood samples, followed by 34% (*n* = 69) for 1000–10,000 and 8% (*n* = 17) for more than 10,000. Only 13% (*n* = 27) of responses were for small sample sizes such as less than 100 samples.

### 3.2 Methods of blood storage used by field biologists

Ethanol, lysis buffer, direct freezing, and filter paper (in decreasing order) were the methods of choice used to store blood samples (Figure 2c). TE buffer, RNA*later*, and other mediums were used in the remaining 16% (44/266) of cases (Figure 2c). In terms of storage temperature, blood samples were stored at −20°C in 34% (62/180) of responses, followed by room temperature, −80°C and +4°C (Figure 2d). Table 1a shows storage methods for blood samples categorized by storage temperature, as reported in the survey.

**TABLE 1:** Number of responses for each type of storage method used, categorized by storage temperature for blood samples (a) and DNA extracts (b).

	RT	+ 4°C	−20°C	−80°C	Other	Total
**(a) Blood samples**						
Ethanol	23 (29)	15 (19)	27 (34)	11 (14)	3 (4)	79
Lysis buffer	14 (29)	11 (22)	12 (24)	9 (18)	3 (6)	49
Direct freezing	0 (0)	0 (0)	18 (43)	22 (52)	2 (5)	42
Filter paper	16 (46)	5 (14)	5 (14)	7 (20)	2 (6)	35
TE buffer	1 (7)	3 (20)	7 (47)	4 (27)	0 (0)	15
RNA*later*	1 (7)	2 (14)	6 (43)	3 (21)	2 (14)	14
Other	0 (0)	2 (17)	3 (25)	6 (50)	1 (8)	12
Total	55	38	78	62	13	246
**(b) DNA extracts**						
TE	0 (0)	2 (3)	60 (80)	13 (17)	0 (0)	75
Water	0 (0)	3 (9)	21 (66)	8 (25)	0 (0)	32
Kit buffer	0 (0)	2 (10)	11 (52)	8 (38)	0 (0)	21
Tris	0 (0)	1 (5)	17 (77)	4 (18)	0 (0)	22
Total	0	8	109	33	0	150

*Note*: Percentages within each storage method are shown in brackets. For blood samples, more than one response could be given. “RT” indicates room temperature. “Other” comprises: EDTA (3 responses), PBS (1), EDTA + PBS (1), a glycerol‐based buffer (1), NBS buffer (1), TNE buffer (2), commercial buffer (3) and heparin buffered tubes (1).

### 3.3 Methods of DNA storage used by field biologists

**FIGURE 3:**
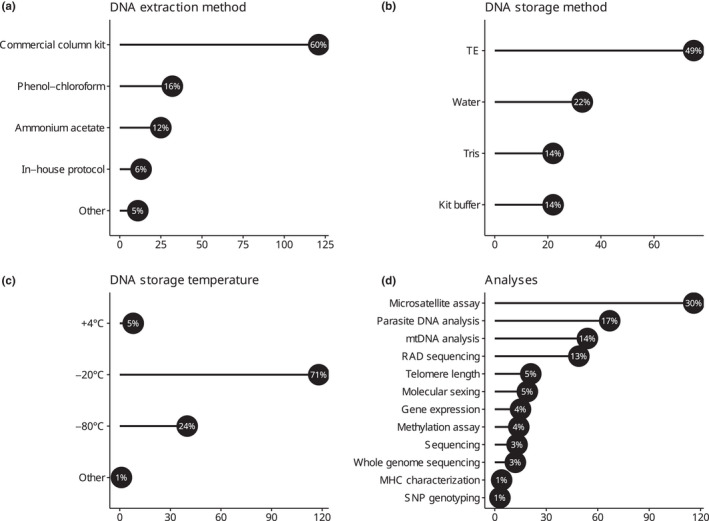
Graphical summary of responses related to: DNA extraction method (a), storage method (b) and storage temperature for DNA extracts (c) and molecular analyses performed by respondents (d). On the *x* axis, numbers of responses, with percentages of the total, are shown; note that in (d) each respondent could provide multiple answers. “Other” in (a) comprises: Commercial magnetic bead kit (3 responses), salt extraction (3), CTAB (1), other types of commercial kits (3) and soda (1). “Other” in (c) refers to −50°C (1 response).

Of the 165 responses to the question “How long after collecting blood samples do you usually extract DNA?,” 26% (*n* = 43) of responses indicated that DNA extraction occurred within 6 months of collection, 19% (*n* = 32) within 1 year of collection, and 19% (*n* = 32) after 1 year. Thirty‐five percent (*n* = 58) of responses were for “I don't have a standard time frame.” Regarding DNA extraction method, 57% (121/214) of responses were for commercial column kit, followed by phenol‐chloroform, ammonium acetate, in‐house protocol, and other methods (Figure 3a). To preserve DNA extracts, TE was most frequently used, followed by water, a kit buffer and Tris (Figure 3b). DNA samples were most frequently archived at −20°C, followed by −80°C and rarely at +4°C (Figure 3c). Table 1b shows storage methods categorized by storage temperature for DNA extracts.

### 3.4 Molecular analyses following DNA extraction

Of the 170 responses to the question “How long after DNA extraction do you usually perform analyses?,” 41% (*n* = 70) were within 6 months of collection, 14% (*n* = 24) within 1 year of collection and 10% (*n* = 17) after 1 year. Thirty‐five percent (*n* = 59) of responses were for “I don't have a standard time frame.” Respondents performed a wide variety of analyses on the collected samples (Figure 3d). Microsatellite assay, parasite DNA screening, mtDNA analysis, and RAD sequencing, in decreasing order, comprised 70% (286/410) of responses. The rest of the answers, in decreasing order, were as follows: SNP chip, telomere length measurement, molecular sexing, gene expression analysis, methylation assay, sequencing, whole‐genome sequencing, MHC characterization, and SNP genotyping (Figure 3d). Table 2 shows the type of molecular analyses performed depending on the type of storage method of blood (a) and DNA (b) samples.

**TABLE 2:** Type of analyses performed on blood (a) and DNA (b),categorized by sample storage method. Percentages within each storage method are shown in brackets. More than one response could be given

	Microsatellite assay	SNP chip	RAD sequencing	Gene expression	Methylation assay	Parasite DNA analysis	mtDNA analysis	Molecular sexing	Sequencing	Telomere length	Whole genome sequencing	SNP genotyping	MHC characterization	Total
**(a) Blood samples**														
Ethanol	53 (27)	12 (6)	25 (13)	4 (2)	5 (3)	35 (18)	29 (15)	10 (5)	6 (3)	10 (5)	6 (3)	1 (1)	1 (1)	197
Lysis buffer	44 (34)	5 (4)	24 (19)	4 (3)	3 (2)	14 (11)	17 (13)	3 (2)	5 (4)	3 (2)	5 (4)	1 (1)	1 (1)	129
Filter paper	26 (29)	4 (4)	15 (16)	1 (1)	3 (3)	9 (10)	19 (21)	6 (7)	2 (2)	1 (1)	3 (3)	1 (1)	1 (1)	91
Direct freezing	15 (19)	3 (4)	8 (10)	2 (2)	3 (4)	17 (21)	9 (11)	6 (8)	3 (4)	10 (12)	2 (2)	2 (2)	0 (0)	80
RNA*later*	8 (20)	3 (7)	3 (7)	5 (12)	1 (2)	9 (22)	6 (15)	0 (0)	1 (2)	2 (5)	1 (2)	0 (0)	2 (5)	41
TE buffer	7 (23)	2 (6)	2 (6)	4 (13)	2 (6)	6 (19)	3 (10)	0 (0)	1 (3)	1 (3)	1 (3)	1 (3)	1 (3)	31
Other	8 (26)	1 (3)	4 (13)	2 (6)	1 (3)	5 (16)	4 (13)	1 (3)	1 (3)	3 (10)	1 (3)	0 (0)	0 (0)	31
Total	161	30	81	22	18	95	87	26	19	30	19	6	6	600
**(b) DNA samples**														
TE	44 (27)	28 (17)	23 (14)	17 (11)	11 (7)	6 (4)	5 (3)	5 (3)	8 (5)	6 (4)	4 (2)	3 (2)	1 (1)	161
Water	18 (23)	15 (19)	14 (18)	13 (16)	0 (0)	5 (6)	2 (3)	1 (1)	3 (4)	2 (3)	5 (6)	0 (0)	1 (1)	79
Kit buffer	12 (28)	5 (12)	6 (14)	4 (9)	4 (9)	3 (7)	3 (7)	4 (9)	0 (0)	0 (0)	1 (2)	1 (2)	0 (0)	43
Tris	13 (30)	9 (21)	2 (5)	7 (16)	2 (5)	2 (5)	4 (9)	2 (5)	0 (0)	2 (5)	0 (0)	0 (0)	0 (0)	43
Total	87	57	45	41	17	16	14	12	11	10	10	4	2	326

### 3.5 Storage issues resulted in low DNA quality and quantity

**FIGURE 4:**
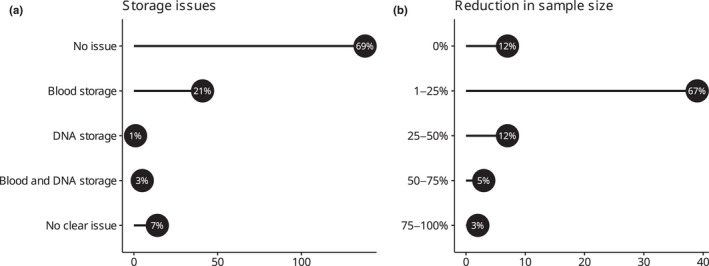
Overview of problems with storage encountered by respondents (a) and reduction in sample size of the project due to storage issues (b). On the *x* axis, numbers of responses, with percentages of the total, are shown

Of the 199 responses to the question on experiencing issues with storage of blood and DNA samples, 31% (*n* = 61) reported problems: 21% (*n* = 41) were problems with storage of blood samples, one with DNA storage and 2% (*n* = 5) with storage of both blood and DNA samples. Seven percent (*n* = 14) of responses were from researchers who experienced problems with sample storage but could not identify the issue (Figure 4a). A total of 44 open responses explained what was the storage issue researchers faced, as follows: (i) use of anticoagulant (possibly due to an overuse of heparin) in collection devices interfering with PCR (5%; *n* = 2), (ii) difficulties with DNA extraction due to lysis buffer, either because of too much blood for the amount of buffer or because of long storage time or lysis buffer interfering with telomere length assay (27%; *n* = 12); (iii) DNA degradation when blood was stored on filter paper, especially in case of long storage time (23%; *n* = 10); (iv) evaporation of ethanol from tubes and ethanol not good enough for PacBio sequencing (20%; *n* = 9), (v) DNA degradation in TE buffer (9%; *n* = 4), (vi) freezer failure (5%; *n* = 2), (vii) misidentification of samples (2%; *n* = 1), (viii) delay of sample shipping (7%; *n* = 3), (ix) difficulties with DNA extraction (2%; *n* = 1). DNA degradation, as reported in the open answers, was not more likely to occur in any of the four storage methods for blood samples indicated by respondents (filter paper, ethanol, lysis buffer, TE buffer; two‐tailed Fisher's exact test, *p*‐value = .136). Of the 61 responses reporting problems with sample storage, 84% (*n* = 51) also reported a reduction in sample size of the project (Figure 4b), which, for example, led to a reduction in the geographic range of the sampling, exclusion of some target species, reduction in statistical power, and left gaps in paternity analyses. Consequently, 18% (*n* = 11) of projects were not published, and 8% (*n* = 5) were published in a less prestigious journal. Storage issues were related to problems with obtaining adequate DNA quality ($\chi_{1}^{2}$ = 28.596, *p*‐value = <.001) and sufficient DNA quantity ($\chi_{1}^{2}$ = 6.139, *p*‐value = .013; Table 3).

**TABLE 3:** Number of responses for problems with sample storage (both blood samples and DNA extracts) with respect to problems with obtaining adequate DNA quality and sufficient DNA quantity. Percentages within rows are shown in brackets.

Storage problems	Problems with DNA quality			Problems with DNA quantity			
	Yes	No	Total	Yes	No	Not measured	Total
Yes	27 (48)	29 (52)	56	19 (35)	32 (59)	3 (6)	54
No	12 (11)	101 (89)	113	18 (16)	82 (73)	13 (11)	113
Total	39	130	169	37	114	16	167

### 3.6 No specific aspect of sample collection and storage influenced DNA quality and quantity

The survey did not identify any association between season when blood samples were collected and problems with obtaining either adequate DNA quality (categories for season: spring, summer, autumn, winter: two‐tailed Fisher's exact test, *p*‐value = .219; categories for season: dry vs. wet season: two‐tailed Fisher's exact test, *p*‐value = .319) or sufficient DNA quantity (categories for season: spring, summer, autumn, winter: two‐tailed Fisher's exact test, *p*‐value = .524; categories for season: dry vs. wet season: two‐tailed Fisher's exact test, *p*‐value = 1). Having access to equipment in the field was not associated with problems with obtaining adequate DNA quality ($\chi_{1}^{2}$ = 0.368, *p*‐value = .544) or sufficient DNA quantity ($\chi_{1}^{2}$ = 0.076, *p*‐value = .783). There was also no association between mode of transferring samples from field to laboratory and problems with obtaining adequate DNA quality (two‐tailed Fisher's exact test, *p*‐value = .160) or sufficient DNA quantity (two‐tailed Fisher's exact test, *p*‐value = .282). No storage method for blood samples was more likely than others to generate problems with obtaining either adequate DNA quality (Table 4a) or sufficient DNA quantity (Table 4b). When researchers were asked whether they changed storage method, a higher number of responses (23%; 43/191) were collected for blood samples compared to DNA extracts (8%; 13/170). Experiencing storage issues was associated with having changed storage method for blood samples ($\chi_{1}^{2}$ = 10.424, *p*‐value = .001). Half (*n* = 8) of the responses explaining why researchers changed storage method for blood samples indicated that the motivation was to increase DNA yields from blood samples and the other half (*n* = 8) reported logistical reasons, either because of space constraints in the laboratory or because of issues during sample transport. There was no association between storage temperature of blood samples and problems with obtaining adequate DNA quality (two‐tailed Fisher's exact test, *p*‐value = .482) or sufficient DNA quantity (two‐tailed Fisher's exact test, *p*‐value = .423). Storage time of blood samples before DNA extraction did not influence either obtaining adequate DNA quality (Table 5a) or sufficient DNA quantity (Table 5b). The DNA extraction method did not predict problems with obtaining either adequate DNA quality (two‐tailed Fisher's exact test, *p*‐value = .268) or sufficient DNA quantity (two‐tailed Fisher's exact test, *p*‐value = .614). In the case of DNA extracts, there was no relationship between storage issues and having changed DNA storage methods (two‐tailed Fisher's exact test, *p*‐value = .210). Among open answers given to explain the change, four reported a change from buffer to water, so the sample was easily concentrated in case of necessity, one a change from −20 to −80°C for logistical reasons, and one because of issues with ethanol. It was not possible to test whether some molecular analysis goals are more sensitive to problems from some storage methods. No molecular analysis was more likely associated with problems with obtaining either adequate DNA quality (two‐tailed Fisher's exact test, *p*‐value = .154) or sufficient DNA quantity (two‐tailed Fisher's exact test, *p*‐value = .871) than others.

**TABLE 4:** Binomial generalized linear models explaining problems with obtaining adequate DNA quality (a) or sufficient DNA quantity (b) based on blood storage method. “Direct freezing” is the reference for parameter estimates.

Variable	Χ^*2*^	Df	Pr(>χ^*2*^)	Estimate ± SE
**(a) DNA quality**	5.703	5, 119	0.3362	
Ethanol				0.821 ± 0.735
Filter paper				0.415 ± 1.006
Lysis buffer				0.128 ± 0.889
Other				0.174 ± 1.262
TE buffer				1.897 ± 0.930
**(b) DNA quantity**	3.292	5, 105	0.655	
Ethanol				0.938 ± 0.758
Filter paper				1.226 ± 0.883
Lysis buffer				0.379 ± 0.844
Other				0.245 ± 1.277
TE buffer				0.091 ± 1.267

**TABLE 5:** Analysis of storage time of blood samples before extraction on problems with obtaining adequate DNA quality (a) or sufficient DNA quantity (b). The analyses (binomial generalized linear models) used the category “Up to 6 months” as reference for parameter estimates.

Variable	χ^*2*^	Df	Pr(>χ^*2*^)	Estimate ± SE
**(a) DNA quality**	1.712	3, 158	0.634	
Up to 1 year				−0.233 ± 0.588
More than 1 year				0.405 ± 0.532
No standard time frame				−0.215 ± 0.500
**(b) DNA quantity**	2.514	3, 141	0.473	
Up to 1 year				0.811 ± 0.609
More than 1 year				0.310 ± 0.651
No standard time frame				0.707 ± 0.550

## 4 DISCUSSION

Proper archiving of biological samples collected in the wild is crucial for current and future research in ecology and evolution, as the way samples are collected and stored has implications for the outcome of the project in many different disciplines. Furthermore, proper archived material provides opportunities for subsequent and future investigations allowed by technical developments (Jackson et al., 2012; Wong et al., 2012). We report first‐hand experiences of ecologists and field biologists regarding worldwide practices for the field collection of blood samples from wild birds and the long‐term storage of blood samples and DNA extracts in the laboratory. We were not able to collect responses to the survey equally around the world, as the majority of participating researchers (85%) were affiliated with institutions in Europe and North America and 63% performed sampling in those continents. Therefore, we obtained limited information for countries and regions of the world where sample collection in the field, temporary field storage and transport to the laboratory might be more challenging from a climatic and/or logistical point of view. For instance, samples collected in Africa, Asia, Oceania, and South America might be more at risk of being compromised due to higher temperatures experienced during sampling in remote locations compared to those in North America and Europe. The collected responses indicated that the blood storage issues and resulting DNA quality/quantity problems were not related to: (i) season of blood sampling, (ii) access to equipment in the field, (iii) means of sample transportation. However, this unequal sampling may have biased our ability to reach conclusions regarding the field collection and transport of blood samples.

In terms of DNA storage, storing conditions most often reported in the present survey overlap with recommendations available in the literature (Table 1; Figure 3; Morin et al., 2010; Prendini et al., 2002). DNA is most often archived at low temperatures (e.g., −20°C) in a neutral pH buffer containing chelating agents such as EDTA (Table 1; Figure 3; Morin et al., 2010; Prendini et al., 2002). In addition, respondents to the survey recommended dividing valuable samples into aliquots, provided space is not an issue, to reduce the risk of damaging DNA when samples are kept in the fridge for a long time or repeatedly frozen and thawed when samples are analyzed. Respondents also shared pros and cons of different extraction methods, based on their experiences: for instance, phenol chloroform is more time consuming and more toxic than commercial column kits, but provides higher DNA yields and is less expensive. Salt precipitation is less toxic, faster, without risk of contaminating samples and gives comparable results in terms of DNA yields relative to phenol‐chloroform. Some respondents also suggested that extracting DNA sooner after collection improved quality. Our results support continued research into extraction techniques that improve processing speed, quality and yield of DNA, and reduce the costs.

While no clear signal emerged from the survey in terms of covariation between specific storage methods and DNA quality and quantity output in downstream lab work, a critical point identified by the survey is that the outcome of molecular analyses often depends on storing conditions of blood samples and less of DNA extracts, possibly because clean DNA is easier to store and/or more resilient to damage. Table 7 integrates personal experiences of respondents with the available literature to provide general recommendations for blood storage and to ameliorate storing practices. For instance, respondents reported that “blood in lysis buffer annoyingly clogs up, whether storing in fridge or freezer” and “freezing often creates a gel‐like consistency that proteases cannot penetrate” (Table 6). By contrast, previous literature recommends storing lysis buffer at +4°C or −20°C (Longmire et al., 1997; Seutin et al., 1991). Surprisingly, according to the survey, there was no indication of storage method or storage temperature for blood samples to affect DNA quality and/or quantity, despite previous studies reporting the opposite and similarly to first‐hand accounts of respondents (Table 6). For instance, storing blood samples on FTA® cards at room temperature was reported to affect RTL measurements compared with frozen blood or DNA (Reichert et al., 2017) and storing blood on Guthrie cards at +4°C affected DNA concentration relative to samples stored at −20°C (Hollegaard et al., 2011). We expected to observe a similar pattern but this was not the case, possibly due to a limited sample size of researchers who had used many different methods. Most respondents also had probably chosen a method most suitable for their research goals. In addition, recent evidence shows that longer storage time as whole blood in ethanol at room temperature has a negative effect on telomere length measurements (Sibma, 2021). Based on the collected responses, there was no indication that storage time had an important influence on DNA quantity or quality. On the contrary, several studies have reported poorer genomic DNA yields during extraction after prolonged storage time of blood samples (Cushwa & Medrano, 1993; Ross et al., 1990; Schröder & Steimer, 2018). Inconsistent results are also available regarding DNA quality: some studies have suggested that genomic DNA extracted after 1 month of storage time provides reliable test results in a number of molecular biology tests (Udtha et al., 2014), while others have observed a decrease in genomic DNA quality after as little as a few days of storage (Malentacchi et al., 2015; Palmirotta et al., 2011). According to our survey, the effectiveness of a preservation method is not the only factor that should be considered when choosing how to collect and store biological material. Our respondents' experiences show that planned or possible future analyses, storage time, logistics in the field, storage space, and sample storage costs all contribute to influence the choice of sample storage conditions. Respondents of the survey also reported that in most cases, it is necessary to find cost‐efficient solutions, often compromising between convenience in the field, storage space in the laboratory and costs for expensive preservation methods or for the maintenance of fridges and freezers. Consequently, logistical or funding reasons might force researchers to adopt more convenient methods that still provide reasonable sample quality for the specific research goal which they originally aimed for. The choices of storage methods are also often based on historical practices; therefore, long‐established methods might often be unsuitable for specific needs, especially for targets (e.g., telomeres) whose importance has emerged more recently. Accordingly, researchers planning new research goals that involve archived samples should make sure that the current storage method and temperature are suitable for the specific target assay, also considering new goals that may emerge in the future. For instance, according to respondents, the quality of DNA extracted from blood samples stored on filter paper was suitable for microsatellites but not for whole‐genome sequencing or RAD sequencing, which require higher quality DNA. For the latter analyses, blood samples should be stored in lysis buffer or ethanol, or even better, frozen or stored in RNA*later*. Moreover, while storing samples in a lysis buffer is more affordable and logistically easier than freezing samples, based on the experience of respondents, it might compromise results of telomere length assays. Lysis buffer samples are also quite sensitive to the quantity of blood used; typically, 20 μl of blood in 1 ml of buffer is a good target, and if more blood is available, placing it in duplicate tubes is preferable. Some of the compromises regarding blood storage may be less detrimental if DNA is extracted as soon as possible, as some respondents reported DNA quality declining with time for some storage methods (lysis buffer or filter paper). These recommendations are targeted to facilitate avoiding problems with sample storage while setting up a long‐term project or while introducing changes in biological sample libraries, possibly because of ineffective DNA preservation. This is of crucial importance, because, as evidenced by the survey, sample storage issues appear central to problems with obtaining adequate DNA quality and sufficient DNA quantity for the intended molecular analyses (Table 3). However, a word of caution is needed here because an inherent limitation of our study is that specific issues might be isolated instances of individual respondents, which may not be shared by the majority of researchers storing the same type of samples in similar conditions or performing the same analyses. This might be the case for some examples shown in Table 6, for instance less than optimal Pac Bio sequencing on blood samples stored in ethanol. From the survey, it is not possible to quantitatively assess the magnitude of similar issues. More formal and targeted studies on limitations of specific storage conditions discussed by respondents will be useful moving forward, in particular for issues for which there are few or no published studies. In addition, technical assessments testing the same issue would benefit from more standard procedures, which would facilitate comparisons among studies and allow to draw more robust generalizations. For instance, inconsistencies among results might derive from the several confounding effects differing among such studies, that is, model organism, additives added to the samples, storage time of blood samples, DNA extraction method and/or yield and quality assessment/quantification, and not from a lack of effect (see Zanet et al., 2013; Reichert et al., 2017 for telomere length; Udtha et al., 2014; Palmirotta et al., 2011 for genomic DNA integrity). Importantly in the context of long‐term field studies, laboratory assessments performed on freshly collected samples might have limited predictive value considering that biological repositories in ecology and evolution require at least 10 or 20 years of monitoring (Mills et al., 2015).

**TABLE 6:** A selection of comments provided by respondents detailing their own problems with specific storage methods and assays. Please note that these opinions are based solely on personal experience and can be treated as case studies/anecdotes for further quantitative investigations rather than systematic recommendations.

**Ethanol**
“While good and sufficient for most things the DNA quality (average fragment size) is too small for optimal Pac Bio sequencing”
“We have never had any problems with genotyping, sex‐typing etc but we now believe that storage of blood in ethanol has a progressive effect on the detection of telomeric sequence by qPCR”
“The blood was put into 100% ethanol and stored long term. These samples were originally collected in 2007 and the tubes must not have been air tight seals as the ethanol evaporated and the blood became dried scabs”
**Lysis buffer**
“It was more difficult to achieve the minimum concentration for RADseq and whole genome with blood samples stored in lysis buffer… sometimes (not always), but increasing the lysis incubation time and eluting with less buffer often did the trick”
“Previous samples were collected into a lysis buffer and stored at RT. Over the years the DNA seems to be of lower quality than that collected recently and stored frozen”
“Used lysis for several years because of ease of preservation, but switched back to freezing when it became apparent this would not work for telomeres”
“Lysis buffers, including “Queen's buffer”, have two serious problems: (1) DNA degrades quickly (potentially within months) if not extracted soon after collection; (2) freezing often creates a gel‐like consistency that proteases cannot penetrate”
**Direct freezing**
“Our freezer failed overnight and so a small number of extracted DNA samples were damaged”
**Filter paper**
“Storage of blood on paper filter during 5 years, sufficient for microsatellite analyses but too degraded for next‐generation sequencing”
“We had issue to perform whole‐genome sequencing from blood samples stored on FTA cards. They generated significantly less DNA and less pair‐ended reads (77 millions vs. up to 215 millions with blood preserved in ethanol). Furthermore, we were not able to generate mate pair libraries out of it because of the lack of DNA available”
“Used to use FTA cards for microsat work, but have since switched to lysis buffer for whole‐genome and RAD sequencing as DNA quality is much higher in buffer compared to on filter paper”

## 5 CONCLUSIONS

Our survey reveals a clear consensus of a compelling need for preserving the biological material that has been collected from wild animals as successfully as possible. The experiences of respondents show that choosing storage method and temperature has the potential to generate considerable variation in DNA quality and/or quantity, with possible nontrivial consequences for research outcomes. We found no perfect method, and the collective experience of the avian researchers' community indicates that multiple factors must be considered when choosing storage conditions (Table 7). For instance, researchers should avoid filter paper for whole‐genome sequencing or RAD sequencing and telomere length measurements, for which frozen samples are the best option. Samples stored in lysis buffer or ethanol may yield satisfactory results with next‐generation sequencing techniques; however, when very high DNA quality is required, it is better to freeze samples or store them in RNA*later*. Other important nuggets of advice include avoiding refrigerating or freezing blood samples stored in lysis buffer; extracting DNA as soon as possible after collection of blood samples, and dividing DNA extracts in aliquots. Depending on research aims, the optimal preservation method should be able to guarantee adequate quality and enough DNA required by the planned assay, but also be flexible enough to offer suitable material for future possible technological developments, as in the case of telomere length measurements. We recommend that researchers setting up or planning to introduce changes in long‐term biological archives carefully take into consideration the effectiveness of currently available preservation methods, together with funding opportunities and logistic limitations.

**TABLE 7:** Storage methods, pros and cons, and recommended best practices for storing blood samples integrated between results of the survey and the available literature

Storage method	Pro	Con	Best practices
Ethanol	Relatively inexpensive, readily available and easy to handle (this study)	Difficult to transport with some shipping companies and evaporation in low‐quality tubes, leading to DNA degradation (this study) Evidence of DNA degradation over long periods of time at room temperature (not specifically blood) (Kilpatrick, 2002)	Optimal concentration between 95%–100% (this study; Wong et al., 2012) Shaking tubes right after collection improves DNA yield (this study) NGS and telomere length measurement may be negatively affected (this study; Sibma, 2021) Remove all ethanol before extraction (this study) Ethanol‐tissue (not specifically blood) ratio at least 3:1 (Wong et al., 2012)
Lysis buffer e.g., Longmire's, Queen's buffer	Relatively inexpensive, easy to handle and transport (this study)	Evidence of DNA degradation over long periods of time at room temperature (this study; Kilpatrick, 2002)	Avoid refrigeration (this study), despite recommendations for storage at +4°C or −20°C (Longmire et al., 1997; Seutin et al., 1991) NGS and telomere length measurement may be negatively affected (this study) Lysis buffer is sensitive to changes in storage temperature (this study) Control amount of blood going into each tube of lysis buffer and collect duplicate tubes (this study) Blood to buffer ratio of 1:10 for Longmire's and Queen's buffer (Longmire et al., 1997; Seutin et al., 1991)
Freezing	−20°C, −80°C or liquid nitrogen provide minimal DNA degradation over long periods of time (Kim et al., 2011)	Difficult access to freezers, dry ice, or liquid nitrogen in remote field locations; difficult shipping of frozen samples; high costs and power consumption; high space requirements and chance of power loss and freeze–thaw cycles (this study)	Works well with all kinds of assays (this study) Setting ULT freezers at −70°C is energy saving compared to −80°C (https://www.freezerchallenge.org/resources.html)
Filter paper	Easy to handle and transport and minimal space requirements (this study)	Relatively expensive (this study) Routinely kept at room temperature, leading to DNA degradation (this study; Carpentieri et al., 2021; Hollegaard et al., 2011)	Long‐term storage should be in a freezer (avoid fridge for risk of developing mildew; this study; Carpentieri et al., 2021; Hollegaard et al., 2011) NGS and telomere length measurement may be negatively affected (this study; Reichert et al., 2017) Extract soon after collection (this study) up to 500 μl maximum total volume/card for Whatman® FTA® card technology (https://www.sigmaaldrich.com/NL/en/substance/whatmanftacardtechnology1234598765?context=product).

## AUTHOR CONTRIBUTIONS

**Irene Di Lecce:** conceptualization (equal); data curation (lead); formal analysis (lead); methodology (equal); writing—original draft (lead); writing—review & editing (equal). **Joanna Sudyka:** conceptualization (equal); methodology (equal); writing—review & editing (equal). **David F. Westneat:** conceptualization (equal); methodology (equal); writing—review & editing (supporting). **Marta Szulkin:** conceptualization (equal); funding acquisition (lead); methodology (equal); writing—review & editing (equal).

## CONFLICT OF INTEREST

We declare none.

## Supporting information


Appendix S1

Appendix S2

Tables S1–S3

## Data Availability

The data that support the findings of this study are openly available at Figshare at https://doi.org/10.6084/m9.figshare.17281793.v1.

## References

[ece39232-bib-0001] Bengston, S. E. , Dahan, R. A. , Donaldson, Z. , Phelps, S. M. , van Oers, K. , Sih, A. , & Bell, A. M. (2018). Genomic tools for behavioural ecologists to understand repeatable individual differences in behaviour. Nature Ecology & Evolution, 2(6), 944–955. 10.1038/s41559-017-0411-4 29434349 PMC9437744

[ece39232-bib-0002] Black, W. C., IV , Baer, C. F. , Antolin, M. F. , & DuTeau, N. M. (2001). Population genomics: Genome‐wide sampling of insect populations. Annual Review of Entomology, 46(1), 441–469. 10.1146/annurev.ento.46.1.441 11112176

[ece39232-bib-0003] Bouwhuis, S. (2018). On the ecological insights provided by a long‐term study on an even longer‐lived bird. Journal of Animal Ecology, 87(4), 891–892. 10.1111/1365-2656.12836 29931771

[ece39232-bib-0004] Carpentieri, D. , Colvard, A. , Petersen, J. , Marsh, W. , David‐Dirgo, V. , Huentelman, M. , Pirrotte, P. , & Sivakumaran, T. A. (2021). Mind the quality gap when banking on dry blood spots. Biopreservation and Biobanking, 19(2), 136–142. 10.1089/bio.2020.0131 33567235

[ece39232-bib-0005] Clutton‐Brock, T. , & Sheldon, B. C. (2010). Individuals and populations: The role of long‐term, individual‐based studies of animals in ecology and evolutionary biology. Trends in Ecology & Evolution, 25(10), 562–573. 10.1016/j.tree.2010.08.002 20828863

[ece39232-bib-0006] Cushwa, W. T. , & Medrano, J. F. (1993). Effects of blood storage time and temperature on DNA yield and quality. BioTechniques, 14(2), 204–207. 8431281

[ece39232-bib-0007] Conrad, K. F. , Robertson, R. J. , & Boag, P. T. (2000). Difficulties storing and preserving tyrant flycatcher blood samples used for genetic analyses. The Condor, 102(1), 191–193. 10.1093/condor/102.1.191

[ece39232-bib-0008] Derrick, B. , & White, P. (2017). Comparing two samples from an individual Likert question. International Journal of Mathematics and Statistics, 18, 1–13.

[ece39232-bib-0009] Deyoung, R. W. , & Honeycutt, R. L. (2005). The molecular toolbox: Genetic techniques in wildlife ecology and management. Journal of Wildlife Management, 69(4), 1362–1384. 10.2193/0022-541X(2005)69[1362:TMTGTI]2.0.CO;2

[ece39232-bib-0010] Freed, L. A. , & Cann, R. L. (2006). DNA quality and accuracy of avian malaria PCR diagnostics: A review. The Condor, 108(2), 459–473.

[ece39232-bib-0012] Hollegaard, M. V. , Grove, J. , Grauholm, J. , Kreiner‐Møller, E. , Bønnelykke, K. , Nørgaard, M. , Benfield, T. L. , Nørgaard‐Pedersen, B. , Mortensen, P. B. , Mors, O. , Sørensen, H. T. , Harboe, Z. B. , Børglum, A. D. , Demontis, D. , Ørntoft, T. F. , Bisgaard, H. , & Hougaard, D. M. (2011). Robustness of genome‐wide scanning using archived dried blood spot samples as a DNA source. BMC Genetics, 12, 58. 10.1186/1471-2156-12-58 21726430 PMC3142526

[ece39232-bib-0555] International Society for Behavioural Ecology (ISBE) . (2018). 17th International Behavioral Ecology Congress. University of Minnesota.

[ece39232-bib-0666] International Ornithological Congress (IOC) . (2018). 27th International Ornithological Congress. Vancouver.

[ece39232-bib-0014] Jackson, J. A. , Laikre, L. , Baker, C. S. , Kendall, K. C. , & The Genetic Monitoring Working Group . (2012). Guidelines for collecting and maintaining archives for genetic monitoring. Conservation Genetics Resources, 4(2), 527–536. 10.1007/s12686-011-9545-x

[ece39232-bib-0015] Jensen, H. , Szulkin, M. , & Slate, J. (2014). Molecular quantitative genetics. In A. Charmantier , D. Garant , & L. E. B. Kruuk (Eds.), Quantitative Genetics in the Wild (pp. 209–227). Oxford University Press.

[ece39232-bib-0016] Kilpatrick, C. W. (2002). Noncryogenic preservation of mammalian tissues for DNA extraction: An assessment of storage methods. Biochemical Genetics, 40, 53–62. 10.1023/A:1014541222816 11989787

[ece39232-bib-0017] Kim, Y.‐T. , Choi, E.‐H. , Son, B.‐K. , Seo, E.‐H. , Lee, E.‐K. , Ryu, J.‐K. , Ha, G.‐W. , Kim, J.‐S. , Kwon, M.‐R. , Nam, J.‐H. , Kim, Y.‐J. , & Lee, K.‐R. (2011). Effects of storage buffer and temperature on the integrity of human DNA. Korean Journal of Clinical Laboratory Science, 44, 24–30.

[ece39232-bib-0018] Longmire, J. , Maltbie, M. , & Baker, R. J. (1997). Use of “lysis buffer” in DNA isolation and its implication for museum collections. Museum of Texas Tech University. 10.5962/bhl.title.143318

[ece39232-bib-0019] Madisen, L. , Hoar, D. I. , Holroyd, C. D. , Crisp, M. , Hodes, M. E. , & Reynolds, J. F. (1987). The effects of storage of blood and isolated DNA on the integrity of DNA. American Journal of Medical Genetics, 27(2), 379–390. 10.1002/ajmg.1320270216 3605221

[ece39232-bib-0020] Malentacchi, F. , Ciniselli, C. M. , Pazzagli, M. , Verderio, P. , Barraud, L. , Hartmann, C. C. , Pizzamiglio, S. , Weisbuch, S. , Wyrich, R. , & Gelmini, S. (2015). Influence of pre‐analytical procedures on genomic DNA integrity in blood samples: The SPIDIA experience. Clinica Chimica Acta, 440, 205–210. 10.1016/j.cca.2014.12.004 25485853

[ece39232-bib-0021] Manel, S. , Berthier, P. , & Luikart, G. (2002). Detecting wildlife poaching: Identifying the origin of individuals with Bayesian assignment tests and multilocus genotypes. Conservation Biology, 16(3), 650–659.

[ece39232-bib-0022] Mei, J. V. , Li, L. , Rasmussen, S. A. , Collier, S. , Frias, J. L. , Honein, M. A. , Shaw, G. M. , Lorey, F. , Meyer, R. , Chaing, S. , Canfield, M. A. , Jones, J. , & Hannon, W. H. (2011). Effect of specimen storage conditions on newborn dried blood spots used to assess *Toxoplasma gondii* immunoglobulin M (IgM). Clinica Chimica Acta, 412(5–6), 455–459. 10.1016/j.cca.2010.11.028 21114968

[ece39232-bib-0023] Michaud, C. L. , & Foran, D. R. (2011). Simplified field preservation of tissues for subsequent DNA analyses*. Journal of Forensic Sciences, 56(4), 846–852. 10.1111/j.1556-4029.2011.01771.x 21480896

[ece39232-bib-0024] Mills, J. A. , Teplitsky, C. , Arroyo, B. , Charmantier, A. , Becker, P. H. , Birkhead, T. R. , Bize, P. , Blumstein, D. T. , Bonenfant, C. , Boutin, S. , Bushuev, A. , Cam, E. , Cockburn, A. , Côté, S. D. , Coulson, J. C. , Daunt, F. , Dingemanse, N. J. , Doligez, B. , Drummond, H. , … Zedrosser, A. (2015). Archiving primary data: Solutions for long‐term studies. Trends in Ecology & Evolution, 30(10), 581–589. 10.1016/j.tree.2015.07.006 26411615

[ece39232-bib-0025] Morin, P. A. , Martien, K. K. , Archer, F. I. , Cipriano, F. , Steel, D. , Jackson, J. , & Taylor, B. L. (2010). Applied conservation genetics and the need for quality control and reporting of genetic data used in fisheries and wildlife management. Journal of Heredity, 101(1), 1–10. 10.1093/jhered/esp107 19959596

[ece39232-bib-0026] Palmirotta, R. , Ludovici, G. , De Marchis, M. L. , Savonarola, A. , Leone, B. , Spila, A. , De Angelis, F. , Della Morte, D. , Ferroni, P. , & Guadagni, F. (2011). Preanalytical procedures for DNA studies: The experience of the interinstitutional multidisciplinary BioBank (BioBIM). Biopreservation and Biobanking, 9(1), 35–45. 10.1089/bio.2010.0027 24850204

[ece39232-bib-0777] Polish Evolutionary Conference (PEC) . (2018). 6th Polish Evolutionary Conference. Warsaw.

[ece39232-bib-0027] Prendini, L. , Hanner, R. , & DeSalle, R. (2002). Obtaining, storing and archiving specimens and tissue samples for use in molecular studies. In R. DeSalle , G. Giribet , & W. Wheeler (Eds.), Techniques in molecular systematics and evolution (pp. 176–248). Birkhäuser.

[ece39232-bib-0028] Primmer, C. R. (2009). From conservation genetics to conservation genomics. Annals of the New York Academy of Sciences, 1162(1), 357–368. 10.1111/j.1749-6632.2009.04444.x 19432656

[ece39232-bib-0029] R Core Team . (2022). R: A language and environment for statistical computing. R Foundation for Statistical Computing. URL. https://www.R‐project.org/

[ece39232-bib-0030] Reichert, S. , Froy, H. , Boner, W. , Burg, T. M. , Daunt, F. , Gillespie, R. , Griffiths, K. , Lewis, S. , Phillips, R. A. , Nussey, D. H. , & Monaghan, P. (2017). Telomere length measurement by qPCR in birds is affected by storage method of blood samples. Oecologia, 184(2), 341–350. 10.1007/s00442-017-3887-3 28547179 PMC5487852

[ece39232-bib-0031] Richardson, A. J. , Narendran, N. , Guymer, R. H. , Vu, H. , & Baird, P. N. (2006). Blood storage at 4°C—Factors involved in DNA yield and quality. Journal of Laboratory and Clinical Medicine, 147(6), 290–294. 10.1016/j.lab.2006.01.005 16750666

[ece39232-bib-0032] Riddle, B. R. , Dawson, M. N. , Hadly, E. A. , Hafner, D. J. , Hickerson, M. J. , Mantooth, S. J. , & Yoder, A. D. (2008). The role of molecular genetics in sculpting the future of integrative biogeography. Progress in Physical Geography: Earth and Environment, 32(2), 173–202. 10.1177/0309133308093822

[ece39232-bib-0033] Ross, K. S. , Haites, N. E. , & Kelly, K. F. (1990). Repeated freezing and thawing of peripheral blood and DNA in suspension: Effects on DNA yield and integrity. Journal of Medical Genetics, 27(9), 569–570. 10.1136/jmg.27.9.569 2231649 PMC1017219

[ece39232-bib-0034] Schröder, C. , & Steimer, W. (2018). gDNA extraction yield and methylation status of blood samples are affected by long‐term storage conditions. PLoS ONE, 13(2), e0192414. 10.1371/journal.pone.0192414 29415017 PMC5802893

[ece39232-bib-0035] Seutin, G. , White, B. N. , & Boag, P. T. (1991). Preservation of avian blood and tissue samples for DNA analyses. Canadian Journal of Zoology, 69(1), 82–90. 10.1139/z91-013

[ece39232-bib-0036] Sibma, A. (2021). A longitudinal analysis of telomeres in an insular house sparrow population (PhD thesis). University of Sheffield.

[ece39232-bib-0037] Smith, L. M. , & Burgoyne, L. A. (2004). Collecting, archiving and processing DNA from wildlife samples using FTA databasing paper. BMC Ecology, 4, 4. 10.1186/1472-6785-4-4 15072582 PMC406513

[ece39232-bib-0038] Stier, A. , Bize, P. , Schull, Q. , Zoll, J. , Singh, F. , Geny, B. , Gros, F. , Royer, C. , Massemin, S. , & Criscuolo, F. (2013). Avian erythrocytes have functional mitochondria, opening novel perspectives for birds as animal models in the study of ageing. Frontiers in Zoology, 10(1), 33. 10.1186/1742-9994-10-33 23758841 PMC3686644

[ece39232-bib-0039] Udtha, M. , Flores, R. , Sanner, J. , Nomie, K. , Backes, E. , Wilbers, L. , & Caldwell, J. (2014). The protection and stabilization of whole blood at room temperature. Biopreservation and Biobanking, 12(5), 332–336. 10.1089/bio.2014.0026 25340942

[ece39232-bib-0040] Visvikis, S. , Schlenck, A. , & Maurice, M. (1998). DNA extraction and stability for epidemiological studies. Clinical Chemistry and Laboratory Medicine, 36(8), 551–555. 10.1515/CCLM.1998.094 9806458

[ece39232-bib-0042] Wong, P. B. , Wiley, E. O. , Johnson, W. E. , Ryder, O. A. , O'Brien, S. J. , Haussler, D. , Koepfli, K. P. , Houck, M. L. , Perelman, P. , Mastromonaco, G. , Bentley, A. C. , Venkatesh, B. , Zhang, Y. P. , Murphy, R. W. , & G10KCOS . (2012). Tissue sampling methods and standards for vertebrate genomics. GigaScience, 1(1), 8. 10.1186/2047-217X-1-8 23587255 PMC3626508

[ece39232-bib-0043] Zanet, D. L. , Saberi, S. , Oliveira, L. , Sattha, B. , Gadawski, I. , & Côté, H. C. (2013). Blood and dried blood spot telomere length measurement by qPCR: Assay considerations. PLoS One, 8(2), e57787. 10.1371/journal.pone.0057787 23451268 PMC3581490

[ece39232-bib-0044] Zimmermann, J. , Hajibabaei, M. , Blackburn, D. C. , Hanken, J. , Cantin, E. , Posfai, J. , & Evans, T. C., Jr. (2008). DNA damage in preserved specimens and tissue samples: A molecular assessment. Frontiers in Zoology, 5(1), 18. 10.1186/1742-9994-5-18 18947416 PMC2579423

