# Supplementary material for: Preserving avian blood and DNA sampled in the wild: A survey of personal experiences
Source: Ecol Evol. 2022 Aug 29;12(8):e9232. doi: 10.1002/ece3.9232 (PMC9424668; doi:10.1002/ece3.9232)
Supplement: Supplementary file 1 — Appendix S1 [file ECE3-12-e9232-s001.pdf]

Preserving avian DNA from the wild: Your experience of blood sampling, DNA extraction and storage

Consent

You are invited to participate in a research study entitled Preserving avian DNA from the wild: Your experience of blood sampling, DNA extraction and storage. This study is carried out by Irene Di Lecce, Joanna Sudyka and Marta Szulkin from the University of Warsaw, Poland, and by David F. Westneat from the University of Kentucky, USA.

The purpose of this survey is to assess common practices used in field collection and storage of avian blood samples and DNA extracts.

This online survey is split into 3 sections, and will take you approximately 15 minutes to complete. Your participation in this study is entirely voluntary and you can withdraw at any time by emailing Irene at *irene.dilecce@cent.uw.edu.pl*. You do not have to answer any questions you do not want to.

If you would like to take the survey multiple times to share your experience from multiple projects, please re-take the survey - such an option will be possible as soon as you finish any round of replies.

We believe there are no known risks associated with this research study; however, as with any online related activity the risk of a breach is always possible. Your responses will be kept strictly confidential, and digital data will be stored in secure computer files. Any report of this research that is made available to the public during seminars, conferences or in publications will not include your name or any other individual information by which you could be identified. In other words, compiled results will be entirely anonymous. If you have questions, fell free to contact Irene at the email address provided above.

Completing the questionnaire below indicates that you are 18 years of age or older, and indicates your consent to participate in this survey.

Preserving avian DNA from the wild: Your experience of blood sampling, DNA extraction and storage

1. Blood sample collection

Please answer questions specifically in the context of one research project you choose to assess in this survey. If you would like to assess multiple research projects you were involved in during your career, you are very welcome to do so: simply re-take the survey - such an option will be possible as soon as you finish any round of replies.

1. What species of bird is the focus of the project you will be reporting on here?

2. Where is the fieldwork on this project carried out? Please indicate location and country.

3. When are blood samples on this project most often collected? Select only one.

- ○ Wet season
- ○ Dry season
- ○ Spring
- ○ Summer
- ○ Autumn
- ○ Winter

4. How are blood samples on this project collected? Select all that apply.

- ☐ Capillary
- ☐ Syringe
- ☐ Microvette
- ☐ Other (please specify)

5. Which laboratory equipment do you use daily during fieldwork for handling blood samples on this project? Select all that apply.

- [ ] Fridge
- [ ] Centrifuge
- [ ] Freezer -20°C
- [ ] Freezer -80°C
- [ ] None
- [ ] Other (please specify)

6. How do you move blood samples from the fieldsite to the permanent laboratory on this project? Select all that apply.

- [ ] Car
- [ ] Train
- [ ] Airplane
- [ ] Courier
- [ ] Other (please specify)

7. Does your shipping strategy (Q6) influence your method of blood sample collection?

- ○ Yes
- ○ No

8. How large is your sample database on this project?

- ○ <100 blood samples
- ○ 101-1,000
- ○ 1,001-10,000
- ○ > 10,000

9. Which genetic analyses are performed on the collected samples? Select all that apply.

- [ ] Microsatellite assay
- [ ] SNP chip
- [ ] RAD sequencing
- [ ] Gene expression
- [ ] Methylation assay
- [ ] Parasite DNA analysis
- [ ] mtDNA analysis
- [ ] Other or more than one (please specify)

10. Have you ever experienced problems in generating data during genetic analyses (Q9) for this project because of storage issues?

- ○ Yes, because of blood storage issues
- ○ Yes, because of DNA storage issues
- ○ Yes, because of both blood and DNA storage issues
- ○ Yes, but not sure what was the issue
- ○ No. You can proceed to Q17

11. If you had problems, what was the issue with a particular method of blood or DNA storage?

12. Did these problems ever reduce the quality of your biological conclusions for the project?

- ○ Yes
- ○ Maybe
- ○ No. You can proceed to Q14

13. How did these problems reduce your biological conclusions?

14. How much did these problems reduce your sample size for analysis? Select one response.

- 0%
- 1-25%
- 25-50%
- 50-75%
- 75-100%

15. Did these problems ever lower the quality of the target journal to which you submitted, or plan to submit, your work on this project?

- Yes
- Maybe
- No

16. Did these problems ever prevent you, or might prevent you, from publishing an aspect of this project?

- Yes
- Maybe
- No

17. Thank you for completing section 1 of this survey! Do you have any further comments?

2. Blood storage

Please answer questions <u>specifically in the context of one research project you choose to assess in this survey</u>. If you would like to assess multiple research projects you were involved in during your career, you are very welcome to do so: simply re-take the survey - such an option will be possible as soon as you finish any round of replies.

18. Which of the following do you use for genetic analyses (Q9) on this project? Select one.

- ◯ Whole blood
- ◯ Red blood cells

19. In what medium do you preserve blood samples for this project? Select all that apply.

- ☐ Ethanol
- ☐ Queen's lysis buffer
- ☐ FTA cards or other filter paper
- ☐ TE buffer
- ☐ RNAlater
- ☐ Direct freezing with no buffer (e.g. in freezer, liquid nitrogen, or dry ice)
- ☐ Other (please specify)

20. Do you use different preservation methods (Q19) for different types of genetic analysis (Q9)?

- ◯ Yes
- ◯ No. You can proceed to Q22

21. Please explain the different preservation methods.

22. At what temperature do you store blood samples? Select one response.

- ◯ Room temperature
- ◯ + 4° C
- ◯ - 20° C
- ◯ - 80° C
- ◯ Other (please specify)

23. Overall, are you satisfied with your current method of preserving blood samples for this project?

- ◯ Very satisfied
- ◯ Satisfied
- ◯ Neither satisfied nor dissatisfied
- ◯ Dissatisfied
- ◯ Very dissatisfied

24. Have you ever changed storage method of blood samples during this project?

- ◯ Yes
- ◯ No. You can proceed to Q26

25. For what reason?

26. Are you currently thinking about changing your storage method for this project?

- ◯ Yes
- ◯ Maybe
- ◯ No. You can proceed to Q28

27. Which storage method are you considering?

28. Would you do something different regarding sample collection or storage method if you had to start your research project all over again?

- ◯ Yes
- ◯ Maybe
- ◯ No. You can proceed to Q30

29. Please explain.

30. Thank you for completing section 2 of this survey! Do you have any further comments?

3. DNA extraction and storage (final section!)

Please answer questions specifically in the context of the research project you choose to assess in this survey. If you would like to assess multiple research projects you were involved in during your career, you are very welcome to do so: simply re-take the survey - such an option will be possible as soon as you finish any round of replies.

31. In which medium do you store extracted DNA for this project?

- Tris
- TE
- Water
- Other (please specify)

32. At what temperature do you store extracted DNA? Select one response.

- + 4° C
- - 20° C
- - 80° C
- Other (please specify)

33. Overall, are you satisfied with your current method of preserving extracted DNA for this project?

- Very satisfied
- Satisfied
- Neither satisfied nor dissatisfied
- Dissatisfied
- Very dissatisfied

34. Have you ever changed storage method of extracted DNA during this project?

- ◯ Yes
- ◯ No. You can proceed to Q36

35. For what reason?

36. Are you currently thinking about changing storage method of extracted DNA for this project?

- ◯ Yes
- ◯ Maybe
- ◯ No. You can proceed to Q38

37. Which storage method are you considering?

38. Have you ever encountered difficulties in obtaining adequate DNA extracts in terms of **quality** because of storage issues?

- ◯ Yes
- ◯ No. You can proceed to Q40

39. What kind of difficulties? Select all that apply.

- ☐ Degraded DNA
- ☐ Impure DNA (poor 260/280 Nanodrop ratio)
- ☐ Impure DNA (poor 230/260 Nanodrop ratio)
- ☐ Impure DNA (poor 260/280 and 230/260 Nanodrop ratios)
- ☐ Other (please specify)

40. Have you ever encountered difficulties in obtaining adequate DNA extracts in terms of **quantity** because of storage issues on this project?

- ◯ Yes
- ◯ No
- ◯ DNA quantity was not measured

41. What kind of DNA extraction protocol do you use for this project?

- ☐ Commercial kit
- ☐ Commercial kit with modified protocol
- ☐ In-house protocol
- ☐ Other (please specify)

42. What kind of DNA extraction method do you use?

- ☐ Chelex
- ☐ Phenol-chloroform
- ☐ Ammonium acetate
- ☐ Other (please specify)

43. Overall, are you satisfied with your current extraction method for this project?

- ◯ Very satisfied
- ◯ Satisfied
- ◯ Neither satisfied nor dissatisfied
- ◯ Dissatisfied
- ◯ Very dissatisfied

44. How long after collecting blood samples do you usually extract DNA?

- ☐ Up to 6 months
- ☐ Up to 1 year
- ☐ More than 1 year
- ☐ I don't have a standard time frame

45. How long after DNA extraction do you usually perfom analyses?

- ☐ Up to 6 months
- ☐ Up to 1 year
- ☐ More than 1 year
- ☐ I don't have a standard time frame

46. Would you do something different regarding the extraction method if you had to start your research project all over again?

- ○ Yes
- ○ Maybe
- ○ No. You can proceed to Q48

47. Please explain.

48. Would you do something different regarding the storage method of extracted DNA if you had to start your research project all over again?

- ○ Yes
- ○ Maybe
- ○ No. You can proceed to Q50

49. Please explain.

50. Based on your experience, did different methods of blood sample storage influence DNA quality or quantity during extraction?

- ○ Yes, DNA quality
- ○ Yes, DNA quantity
- ○ Don't know. You can proceed to Q52
- ○ No. You can proceed to Q52

51. Can you say how?

52. Thank you for completing section 3 of this survey! Do you have any further comments?

THANK YOU for participating!

53. Basic information about you

Name & Surname

Research Institution

City/Town

Country

Work Email Address

You are done! Thank you for participating - we believe that a global assessment of how avian researchers store their blood samples and DNA extracts, and an account of the adversities they have faced when extracting DNA for genetic analyses is greatly needed. We hope that quantitative and qualitative data derived from this survey can allow us to identify whether any procedural or methodological gaps exist - an information that is often anecdotal when the experiences of only a few researchers from molecular ecology labs are compared.

We hope that the results of this survey and the experiences shared by its participants will help improving existing guidelines relevant for the long-term storage of biological data - we will aim to share our conclusions from this survey as soon as possible.

Sincerely,
Irene Di Lecce, Joanna Sudyka, David F. Westneat and Marta Szulkin
