# Supplementary material for: Preserving avian blood and DNA sampled in the wild: A survey of personal experiences
Source: Ecol Evol. 2022 Aug 29;12(8):e9232. doi: 10.1002/ece3.9232 (PMC9424668; doi:10.1002/ece3.9232)
Supplement: Supplementary file 3 — Tables S1–S3 [file ECE3-12-e9232-s003.docx]

Table S1 – List of species sampled by respondents

|  | Common name | Latin/Scientific name | Number of responses |
| --- | --- | --- | --- |
| 1 | Blue tit | *Cyanistes caeruleus* | 17 |
| 2 | Great tit | *Parus major* | 15 |
| 3 | Rock dove | *Columba livia* | 6 |
| 4 | Black-legged kittiwake | *Rissa tridactyla* | 4 |
| 5 | Collared flycatcher | *Ficedula albicollis* | 4 |
| 6 | Common starling | *Sturnus vulgaris* | 4 |
| 7 | House sparrow | *Passer domesticus* | 4 |
| 8 | Mallard | *Anas platyrhynchos* | 4 |
| 9 | Pied flycatcher | *Ficedula hypoleuca* | 4 |
| 10 | Tree swallow | *Tachycineta bicolor* | 4 |
| 11 | Adélie penguin | *Pygoscelis adeliae* | 3 |
| 12 | Black-capped chickadee | *Poecile atricapillus* | 3 |
| 13 | Eurasian blackcap | *Sylvia atricapilla* | 3 |
| 14 | European blackbird | *Turdus merula* | 3 |
| 15 | Red-winged blackbird | *Agelaius phoeniceus* | 3 |
| 16 | Thick-billed murre | *Uria lomvia* | 3 |
| 17 | Alpine swift | *Tachymarptis melba* | 2 |
| 18 | Barn swallow | *Hirundo rustica* | 2 |
| 19 | Berthelot's pipit | *Anthus berthelotii* | 2 |
| 20 | Blue-black grassquit | *Volatinia jacarina* | 2 |
| 21 | Blue-footed booby | *Sula nebouxii* | 2 |
| 22 | Carolina chickadee | *Poecile carolinensis* | 2 |
| 23 | Dark-eyed junco | *Junco hyemalis* | 2 |
| 24 | Eastern bluebird | *Sialia sialis* | 2 |
| 25 | Little auk | *Alle alle* | 2 |
| 26 | Mountain chickadee | *Poecile gambeli* | 2 |
| 27 | Peregrine falcon | *Falco peregrinus* | 2 |
| 28 | Red kite | *Milvus milvus* | 2 |
| 29 | Red-backed fairywren | *Malurus melanocephalus* | 2 |
| 30 | Rufous-collared sparrow | *Zonotrichia capensis* | 2 |
| 31 | Savannah sparrow | *Passerculus sandwichensis* | 2 |
| 32 | Seychelles warbler | *Acrocephalus sechellensis* | 2 |
| 33 | Spotless starling | *Sturnus unicolor* | 2 |
| 34 | White-throated sparrow | *Zonotrichia albicollis* | 2 |
| 35 | Zebra finch | *Taeniopygia guttata* | 2 |
| 36 | African penguin | *Spheniscus demersus* | 1 |
| 37 | American kestrel | *Falco sparverius* | 1 |
| 38 | Andean condor | *Vultur gryphus* | 1 |
| 39 | Asian Houbara bustard | *Chlamydotis macqueenii* | 1 |
| 40 | Bachman’s sparrow | *Peucaea aestivalis* | 1 |
| 41 | Bahama oriole | *Icterus northropi* | 1 |
| 42 | Bar-tailed godwit | *Limosa lapponica baueri* | 1 |
| 43 | Barred owl | *Strix varia* | 1 |
| 44 | Bearded reedling | *Panurus biarmicus* | 1 |
| 45 | Black grouse | *Lyrurus tetrix* | 1 |
| 46 | Black guillemot | *Cepphus grylle* | 1 |
| 47 | Black sparrowhawk | *Accipiter melanoleucus* | 1 |
| 48 | Blue crane | *Grus paradisea* | 1 |
| 49 | Brown booby | *Sula leucogaster* | 1 |
| 50 | Bulwer's petrel | *Bulweria bulwerii* | 1 |
| 51 | Cape vulture | *Gyps coprotheres* | 1 |
| 52 | Chinstrap penguin | *Pygoscelis antarcticus* | 1 |
| 53 | Cliff swallow | *Petrochelidon pyrrhonota* | 1 |
| 54 | Common linnet | *Linaria cannabina* | 1 |
| 55 | Common murre | *Uria aalge* | 1 |
| 56 | Common redstart | *Phoenicurus phoenicurus* | 1 |
| 57 | Common whitethroat | *Sylvia communis* | 1 |
| 58 | Corncrake | *Crex crex* | 1 |
| 59 | Cory's shearwater | *Calonectris borealis* | 1 |
| 60 | Dunnock | *Prunella modularis* | 1 |
| 61 | Eurasian reed warbler | *Acrocephalus scirpaceus* | 1 |
| 62 | Eurasian stone-curlew | *Burhinus oedicnemus* | 1 |
| 63 | European turtle dove | *Streptopelia turtur* | 1 |
| 64 | European woodcock | *Scolopax rusticola* | 1 |
| 65 | Field sparrow | *Spizella pusilla* | 1 |
| 66 | Florida scrub jay | *Aphelocoma coerulescens* | 1 |
| 67 | Galapagos mockingbird | *Mimus parvulus* | 1 |
| 68 | Gentoo penguin | *Pygoscelis papua* | 1 |
| 69 | Great dusky swift | *Cypseloides senex* | 1 |
| 70 | Great-tailed grackle | *Quiscalus mexicanus* | 1 |
| 71 | Greater ani | *Crotophaga major* | 1 |
| 72 | Greater flamingo | *Phoenicopterus roseus* | 1 |
| 73 | Grey crowned crane | *Balearica regulorum* | 1 |
| 74 | Gyrfalcon | *Falco rusticolus* | 1 |
| 75 | Hawaiian goose | *Branta sandvicensis* | 1 |
| 76 | Herring gull | *Larus argentatus* | 1 |
| 77 | Hihi | *Notiomystis cincta* | 1 |
| 78 | House finch | *Haemorhous mexicanus* | 1 |
| 79 | Ivory gull | *Pagophila eburnea* | 1 |
| 80 | King penguin | *Aptenodytes patagonicus* | 1 |
| 81 | Lappet-faced vulture | *Torgos tracheliotos* | 1 |
| 82 | Leach's storm petrel | *Oceanodroma leuchoroa* | 1 |
| 83 | Magpie | *Pica pica* | 1 |
| 84 | Marsh tit | *Poecile palustris* | 1 |
| 85 | Mauritius parakeet | *Psittacula eques* | 1 |
| 86 | Monteiro's storm petrel | *Oceanodroma monteiroi* | 1 |
| 87 | North African Houbara bustard | *Chlamydotis undulata undulata* | 1 |
| 88 | Northern cardinal | *Cardinalis cardinalis* | 1 |
| 89 | Northern flicker | *Colaptes auratus* | 1 |
| 90 | Ocellated turkey | *Meleagris ocellata* | 1 |
| 91 | Red-eyed vireo | *Vireo olivaceus* | 1 |
| 92 | Red-throated caracara | *Ibycter americanus* | 1 |
| 93 | Saltmarsh sparrow | *Ammospiza caudacuta* | 1 |
| 94 | Sand martin | *Riparia riparia* | 1 |
| 95 | Seaside sparrow | *Ammospiza maritima* | 1 |
| 96 | Sedge warbler | *Acrocephalus schoenobaenus* | 1 |
| 97 | Silvereye | *Zosterops lateralis* | 1 |
| 98 | Song sparrow | *Melospiza melodia* | 1 |
| 99 | Sooty swift | *Cypseloides fumigatus* | 1 |
| 100 | Southern crested caracara | *Caracara plancus* | 1 |
| 101 | Southern dunlin | *Calidris alpina schinzii* | 1 |
| 102 | Southern pied babbler | *Turdoides bicolor* | 1 |
| 103 | Spotted owl | *Strix occidentalis* | 1 |
| 104 | Sun parakeet | *Aratinga solstitialis* | 1 |
| 105 | Superb fairywren | *Malurus cyaneus* | 1 |
| 106 | Swainson's hawk | *Buteo swainsoni* | 1 |
| 107 | Tawny owl | *Strix aluco* | 1 |
| 108 | Wattled crane | *Grus carunculata* | 1 |
| 109 | Western bluebird | *Sialia mexicana* | 1 |
| 110 | Western capercaillie | *Tetrao urogallus* | 1 |
| 111 | Western sandpiper | *Calidris mauri* | 1 |
| 112 | Whimbrel | *Numenius phaeopus* | 1 |
| 113 | Whinchat | *Saxicola rubetra* | 1 |
| 114 | Whiskered tern | *Chlidonias hybrida* | 1 |
| 115 | White stork | *Ciconia ciconia* | 1 |
| 116 | White-backed vulture | *Gyps africanus* | 1 |
| 117 | White-browed sparrow-weaver | *Plocepasser mahali* | 1 |
| 118 | White-collared swift | *Streptoprocne zonaris* | 1 |
| 119 | White-winged snowfinch | *Montifringilla nivalis* | 1 |
| 120 | Wire-tailed manakin | *Pipra filicauda* | 1 |
| 121 | Wood duck | *Aix sponsa* | 1 |
| 122 | Wood warbler | *Phylloscopus sibilatrix* | 1 |
| 123 | Yellow-legged gull | *Larus michahellis* | 1 |

Table S2 – List of families sampled by respondents

|  | Family name | Number of responses |
| --- | --- | --- |
| 1 | Paridae | 41 |
| 2 | Passerellidae | 13 |
| 3 | Muscicapidae | 10 |
| 4 | Accipitridae | 9 |
| 5 | Hirundinidae | 9 |
| 6 | Laridae | 9 |
| 7 | Alcidae | 7 |
| 8 | Columbidae | 7 |
| 9 | Falconidae | 7 |
| 10 | Spheniscidae | 7 |
| 11 | Anatidae | 6 |
| 12 | Passeridae | 6 |
| 13 | Scolopacidae | 6 |
| 14 | Sturnidae | 6 |
| 15 | Turdidae | 6 |
| 16 | Acrocephalidae | 5 |
| 17 | Apodidae | 5 |
| 18 | Icteridae | 5 |
| 19 | Maluridae | 4 |
| 20 | Sylviidae | 4 |
| 21 | Gruidae | 3 |
| 22 | Phasianidae | 3 |
| 23 | Strigidae | 3 |
| 24 | Sulidae | 3 |
| 25 | Thraupidae | 3 |
| 26 | Cathartidae | 2 |
| 27 | Corvidae | 2 |
| 28 | Estrildidae | 2 |
| 29 | Fringillidae | 2 |
| 30 | Hydrobatidae | 2 |
| 31 | Motacillidae | 2 |
| 32 | Otididae | 2 |
| 33 | Procellaridae | 2 |
| 34 | Zosteropidae | 2 |
| 35 | Burhinidae | 1 |
| 36 | Cardinalidae | 1 |
| 37 | Charadriidae | 1 |
| 38 | Ciconiidae | 1 |
| 39 | Cuculidae | 1 |
| 40 | Leiothrichidae | 1 |
| 41 | Mimidae | 1 |
| 42 | Notiomystidae | 1 |
| 43 | Panuridae | 1 |
| 44 | Phoenicopteridae | 1 |
| 45 | Phylloscopidae | 1 |
| 46 | Picidae | 1 |
| 47 | Pipridae | 1 |
| 48 | Ploceidae | 1 |
| 49 | Prunellidae | 1 |
| 50 | Psittacidae | 1 |
| 51 | Psittaculidae | 1 |
| 52 | Rallidae | 1 |
| 53 | Vireonidae | 1 |

Table S3 – List of orders sampled by respondents

|  | Order name | Number of responses |
| --- | --- | --- |
| 1 | Passeriformes | 144 |
| 2 | Charadriiformes | 26 |
| 3 | Accipitriformes | 10 |
| 4 | Columbiformes | 8 |
| 5 | Falconiformes | 8 |
| 6 | Sphenisciformes | 7 |
| 7 | Anseriformes | 6 |
| 8 | Galliformes | 5 |
| 9 | Apodiformes | 4 |
| 10 | Procellariformes | 4 |
| 11 | Gruiformes | 3 |
| 12 | Strigiformes | 3 |
| 13 | Suliformes | 3 |
| 14 | Otidiformes | 2 |
| 15 | Psittaciformes | 2 |
| 16 | Cathartiformes | 1 |
| 17 | Ciconiiformes | 1 |
| 18 | Cuculiformes | 1 |
| 19 | Phoenicopteriformes | 1 |
| 20 | Piciformes | 1 |
